# High Medication Non-Adherence Rates and its Drivers in the General Population: A Cross-sectional Study Using the OMAS-37 Adherence Survey Tool

**DOI:** 10.1177/00469580251321596

**Published:** 2025-02-24

**Authors:** Rønnaug Eline Larsen, Ala Karimi, Tonje Krogstad, Cecilie Johannessen Landmark, Lene Berge Holm

**Affiliations:** 1 Deparment of Pharmacy, Faculty of Health Sciences, Oslo Metropolitan University, Oslo, Norway; 2 Section for Clinical Pharmacology, SSE, Department of Pharmacology, Oslo University Hospital, Oslo, Norway; 3 The Health Services Recearh Unit, HØKH, Akershus University Hospital, Lørenskog, Norway

**Keywords:** Medication adherence, non adherence, OMAS-37, medication safety, patient-reported outcome measure, cross-sectional studies, shared decision making

## Abstract

Substantial variability in patients’ medication adherence underscores the key significance of pharmacists and other healthcare providers proactively aiding individuals in achieving optimal medication outcomes. Medication-taking behaviours, barriers, and beliefs varies significantly among medication users. It is crucial to ascertain these factors when designing adherence interventions. The OsloMet Adherence-to-medication Survey tool (OMAS-37) is designed to quantify the degree of adherence, and to assess 37 unique causes for non-adherence. The aim of this study was to assess non-adherence among medication users in the general population utilising the OMAS-37 tool. A cross sectional study among medication users in Norway was conducted in 2021. The features of the general population and three patient subgroups—cardiovascular, pain, and mental health disorders—were characterized and compared using Kruskal-Wallis tests. Of the 812 participants, with a median age of 50 (IQR 37-59) and 91% (n = 736) identifying as female, 64% (n = 517) exhibited high non-adherence scores indicating poor medication. Main reasons included forgetfulness (42%, n = 343), perceived improvement in health (40%, n = 326), and fear of adverse drug reactions (39%, n = 320). Statistically significant positive adherence factors encompassed increasing age, higher education, medication decision involvement, and pill organiser usage. The cardiovascular subgroup exhibited significantly better adherence than the pain and mental health disorders subgroups. The total sample and all three subgroups demonstrated some variation in the main causes for non-adherence. This first study employing OMAS-37 reveals high non-adherence levels and varying causes of non-adherence among different patient groups, emphasizing the need for targeted adherence interventions.

**Highlights**

● A Majority (64%) of medication users exhibited high non-adherence, with forgetfulness, perceived health improvement, and fear of adverse drug reactions being the most common reasons.

● Better adherence was associated with increasing age, higher education, involvement in medication decisions, and use of a pill organizer.

● Cardiovascular patients demonstrated significantly better adherence compared to those with pain or mental health disorders.

● Non-adherence causes varied across patient groups, with fear of adverse drug reactions ranking highest in the pain group and forgetfulness being most prominent in the mental health and cardiovascular groups.

● The OsloMet Adherence-to-Medication Survey Tool (OMAS-37) proved useful in quantifying adherence levels and identifying patient-specific barriers to adherence.

## Introduction

It is widely known that medication adherence rates varies, and this underscores the crucial role of healthcare providers in actively supporting patients to ensure optimal medication outcomes, promoting patient safety and enhancing healthcare quality.^1,2^ Adherence is influenced by various patient-related factors, such as forgetfulness and fear of adverse effects, as well as the impact of the medical condition itself.^2,3^ Varying adherence levels to the same medical conditions are evident, suggesting the influence of confounding factors such as drug treatment duration and sociodemographic variables.^2-5^ Contrary to the common belief that older persons have poor adherence, several studies indicate that adherence tends to increase with age, with young individuals being among the most non-adherent.^6,7^

Incorporating patients into the decision-making process concerning their drug therapy also appears to have a positive impact on adherence.^2,8^

Knowledge is scarce regarding rates and causes of non-adherence in the general Norwegian population. Recent studies on non-adherence primarily examine adherence rates for specific medications and conditions (adherence percentages in brackets), such as cardiovascular medications (84-55%),^9,10^ or medications for pregnancy (13%), breastfeeding (38%)^11,12^ and treatment of epilepsy (81%-60%).^13,14^ However, none of these studies quantifies the causes of non-adherence. Although several validated self-reporting adherence scales exist, few provide comprehensive insight into non-adherence causes, often focusing on either medication-taking behaviours and barriers, or beliefs concerning the condition and medication. It is a challenge to reach out to large patient populations and to cover all aspects of adherence in single studies with existing adherence measuring tools. The newly validated OsloMet Adherence-to-medication Survey-tool (OMAS-37) can be utilised to evaluate adherence both in the general population and in various patient groups. This self-reporting adherence tool is designed to assess 37 unique causes of non-adherence related to medication-taking behaviours, barriers, and beliefs.^15^

The aim of this study was to evaluate the usefulness of OMAS-37 in identifying non-adherence rates, both in the general population and in specific patient groups. Additionally, the study sought to examine the distinct causes of suboptimal adherence in different patient groups, which is a crucial aspect for informing the design of future adherence-promoting interventions.

## Method

### Study design and data collection

The data for this cross-sectional study were collected between April 1st and August 1st, 2021, using an OMAS-37-incorporated-survey. (The survey is available in the supplementary material accompanying this article). The OMAS-37 is a valid and reliable instrument, published in 2022, with a Cronbach’s alpha = 0.91.^15^ This cross-sectional study utilises the same data collected for the validation publication; however, a slightly smaller subset of the data is used in this study.^15^ The study included participants aged 18 years or older who resided in Norway and had used medication prescribed or recommended by a physician within the past 12 months. This includes both patients on regular regimens (daily, weekly, or other dosing intervals) and those using medications sporadically, such as for example antibiotics or seasonal allergy treatments. Individuals who reported being under 18, not having used such medications in the last year, or not living in Norway were excluded from the survey and redirected before reaching the questionnaire items.

The OMAS-37 tool assesses 37 distinct causes of medication non-adherence and is designed for both quantifying the overall degree of non-adherence and evaluating the relative importance of each cause. The total non-adherence score is calculated as the sum of scores across all 37 items, ranging from 0 to 111, with higher scores indicating greater levels of non-adherence. In this study, a clinical cut-off score of 2 was applied: scores of 0 or 1 signify good adherence, while scores of 2 or higher denote poor adherence. For a detailed description of the OMAS-37 survey tool, please refer to the original validation publication.^15^

The e-survey also included questions regarding demographics, medical conditions, and medication usage. Respondents were asked to indicate the medical conditions for which they received medication within the last 12 months, giving respondents the choice to select one condition group or more from 24 medical condition groups. The e-survey also included an anchor-question to assess respondents’ beliefs about their adherence relative to their non-adherence score, and a shared decision making (SDM)-question to measure respondents’ sense of inclusion in the decision-making process regarding their medication treatment. To ensure completeness of responses, all questions were mandatory. Furthermore, the e-survey incorporated adaptive features to exclude respondents not meeting the predefined inclusion criteria. Prior to submission, respondents were afforded the option to navigate back to prior pages by selecting “Previous page.”

Participants were recruited from six large Norwegian Facebook groups that encompassed both health and non-health related subjects: *The Public Health Association* (2,3k members/followers), *No to the closure of Ullevaal hospital* (32.2k), *Dental treatment/finances* (80k), *Help on everything in Norway* (33k), *Focus on fibromyalgia* (9.4k), and *Home remedy* (57k). The e-survey announcement was posted on these pages as a voluntary open survey after obtaining moderator approval. The e-survey link was active throughout the entire data collection period. Furthermore, 217 participants were recruited by posting the survey announcement on the Facebook pages of 6 pharmacy students, and data from 39 participants across three pilot studies using OMAS-37 were incorporated. The e-survey announcement is accessible in the supplementary materials.

The data used in this publication were anonymized, and written consent was not sought from the respondents of this questionnaire. Ethical approval was not obtained for this specific data collection, but it was obtained for the overall research project that this publication is a part of. The overall research project received approval from the Norwegian Centre for Research Data (NSD), now renamed the Norwegian Agency for Shared Services in Education and Research (SIKT, reference 479481), and the Regional Committees for Medical and Health Research Ethics (REK, reference 270146).

### Statistical data analysis

Descriptive statistics were used on demographics, medical conditions, and medication usage data from all 812 respondents. Non-adherence scores revealed non-normal distribution with a negative exponential form. Kruskal-Wallis tests were conducted to evaluate the association between different independent variables on non-adherence scores. The *p*-values from these tests were compared to a significance level (α) of 0.05. For variables with *p* < 0.05 in the Kruskal-Wallis tests, pairwise post hoc comparisons were automatically performed for the categories of the independent variable. To account for multiple testing in these pairwise comparisons, Bonferroni procedures were applied, and adjusted p-values were calculated. Results with adjusted p-values below 0.05 were considered statistically significant. All independent variables are listed in the results section. Kruskal-Wallis tests were also employed to compare non-adherence scores among three patient subgroups who had received medication for pain, cardiovascular diseases (CVD) and mental health disorders (MHD) within the past 12 months. The three patient subgroups were chosen for comparison as examples to identify potential differences among various medical condition subgroups.

Pain, CVD and MHD were selected by 551 respondents, with 200 of them reporting more than one of these three conditions. For between-groups analysis, these overlapping respondents were excluded, leaving a final selection of 351 respondents: CVD n = 117, pain n = 190 and MHD n = 44.

All data were analysed by SPSS Statistics version 27 and Microsoft 365 Excel version 2208. The selected significance level alpha was 0.05. The results are reported in accordance with the Checklist for Reporting Results of Internet E-Surveys (CHERRIES).^16^

## Results

### Demographic and medication regime profile

**Figure 1.**
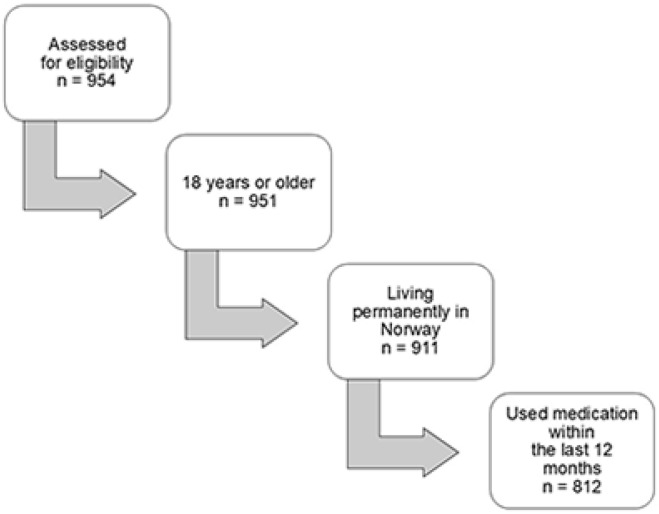
Respondent flow-chart of assessment for eligibility.

The e-survey was completed by 954 respondents, of whom 812 met the eligibility criteria (Figure 1). The demographics and medication usage profile of the 812 respondents is presented in Table 1. Most respondents identified as female (90.6%, n = 736), with a median age of 50 (IQR 37-59). Respondents reported a high level of involvement in their medication regimen; with over 99% (n = 806) self-administering their medications. As indicated by their responses to the SDM-question, 84.6% (n = 687) expressed that they were “largely” or “very largely” involved in decisions regarding their medication treatment. In response to the anchor-question, 96.1% (780) of the total sample believed that they were adhering to their doctor’s medication recommendations to a “large extent” or “very large extent”. This also applies to the three patient subgroups. However, in the CVD-group, more respondents chose “to a large extent” compared to the total sample and the other subgroups.

**Table 1.** Demographic and medication usage profiles for the total sample (total) and the patient subgroups Mental health disorders (MHD), pain and cardiovascular diseases (CVD).

Sample		Total n = 812 (100%)	MHD n = 154 (100%)	Pain n = 365 (100%)	CVD n = 257 (100%)
Age	Median	50.0 years	48.0 years	48.0 years	59.0 years
	Mean	48.0 years	45.9 years	46.7 years	58.3 years
	SD Mean	15.2	13.0	13.2	11.6
	Young 18-44 years	303 (37.3)	64 (41.6)	148 (40.5)	28 (10.9)
	Middle aged 45-65 years	404 (49.8)	81 (52.6)	190 (52.1)	159 (61.9)
	Young-old 66-79 years	99 (12.2)	9 (5.8)	26 (7.1)	66 (25.7)
	Old-old 80-89 years	6 (0.7)	0	1 (0.3)	4 (1.6)
Gender	Female	736 (90.6)	135 (87.7)	344 (94.2)	221 (86.0)
	Male	71 (8.7)	16 (10.4)	21 (5.8)	36 (14.0)
	Do not know or want to tell/NA^a^	5 (0.6)	3 (1.9)	0	0
Born and raised	In Norway	748 (92.1)	142 (92.2)	342 (93.7)	245 (95.3)
	Born abroad, but lived more than eight years	30 (3.7)	5 (3.2)	7 (1.9)	5 (1.9)
	between ages 0 and 18 in Norway				
	Outside Norway	33 (4.1)	7 (4.5)	15 (4.1)	7 (2.7)
	Do not know or want to tell/NA^a^	1 (0.1)	0	1 (0.3)	0
Education level	No education	7 (0.9)	2 (1.3)	4 (1.1)	1 (0.4)
	Primary school only	81 (10.0)	24 (15.6)	42 (11.5)	26 (10.1)
	High school or equivalent	425 (52.3)	81 (52.6)	185 (50.7)	136 (52.9)
	Bachelor’s degree or equivalent	193 (23.8)	36 (23.4)	98 (26.8)	53 (20.6)
	Master’s degree or equivalent	96 (11.8)	10 (6.5)	33 (9.1)	35 (13.6)
	Do not know or want to tell/NA^a^	10 (1.2)	1 (0.6)	3 (0.8)	6 (2.3)
Number of selected medical condition groups^b^	1	164 (20.2)	9 (5.8)	28 (7.7)	27 (10.5)
	2	184 (22.7)	21 (13.6)	54 (14.8)	36 (14.1)
	3	163 (20.1)	28 (18.2)	76 (20.8)	50 (19.5)
	4	102 (12.6)	27 (17.5)	58 (15.9)	33 (12.9)
	5-9	185 (22.8)	64 (41.6)	137 (37.5)	101 (39.5)
	10-13	14 (1.7)	5 (3.2)	12 (3.3)	10 (3.9)
Years of regular medication use	0-1	53 (6.5)	12 (7.8)	19 (5.2)	6 (2.3)
	2-5	146 (18.0)	22 (14.3)	61 (16.7)	25 (9.7)
	6-9	109 (13.4)	19 (12.3)	37 (10.1)	36 (14.0)
	10 or more	444 (54.7)	95 (61.7)	223 (61.1)	185 (72.0)
	Not using regularly	49 (6.0)	3 (1.9)	20 (5.5)	1 (0.4)
	Do not know or want to tell/NA^a^	11 (1.4)	3 (1.9)	5 (1.4)	4 (1.6)
Number of daily medications	0	52 (6.4)	3 (1.9)	23 (6.3)	2 (0.8)
	1	146 (18.0)	12 (7.8)	49 (13.4)	16 (6.2)
	2	150 (18.5)	25 (16.2)	61 (16.7)	23 (8.9)
	3	138 (17.0)	27 (17.5)	65 (17.8)	37 (14.4)
	4	97 (11.9)	19 (12.3)	40 (11.0)	40 (15.6)
	5-9	166 (20.4)	49 (31.8)	88 (24.1)	99 (38.5)
	10 or more	55 (6.8)	17 (11)	36 (9.9)	39 (15.2)
	Do not know or want to tell/NA^a^	8 (1.0)	2 (1.3)	3 (0.8)	2 (0.8)
Using a Pill Organiser	Yes	319 (39.3)	85 (55.2)	158 (43.3)	149 (58)
	No	485 (59.7)	68 (44.2)	202 (55.3)	107 (41.6)
	Do not know or want to tell/NA^a^	8 (1.0)	1 (0.6)	5 (1.4)	1 (0.4)
Are mainly responsible for taking the medicines themselves	Yes	806 (99.3)	152 (98.7)	363 (99.5)	255 (99.2)
	No	6 (0.7)	2 (1.3)	2 (0.5)	2 (0.8)
Anchor-question: To what extent they believe they are following the recommendations from their doctor regarding their medication use	To a very large extent	574 (70.7)	108 (70.1)	250 (68.5)	214 (83.3)
	To a large extent	206 (25.4)	40 (26)	101 (27.7)	38 (14.8)
	To a limited extent	24 (3.0)	4 (2.6)	10 (2.7)	3 (1.2)
	To a very limited extent	5 (0.6)	1 (0.6)	3 (0.8)	1 (0.4)
	Do not know or want to tell/NA^a^	3 (0.4)	1 (0.6)	1 (0.3)	1 (0.4)
SDM-question: To what extent they feel involved in the decisions made regarding their medication treatment	To a very large extent	418 (51.5)	84 (54.5)	185 (50.7)	142 (55.3)
	To a large extent	269 (33.1)	49 (31.8)	125 (34.2)	84 (32.7)
	To a limited extent	84 (10.3)	12 (7.8)	36 (9.9)	25 (9.7)
	To a very limited extent	20 (2.5)	5 (3.2)	8 (2.3)	5 (2)
	Do not know or want to tell/NA^a^	21 (2.6)	4 (2.6)	11 (3)	1 (0.4)

a NA; Not applicable.

b Could select 1 or more from 24 different medical condition groups.

### Adherence measurement

The median total non-adherence score was 3 (IQR 0-10). The most common scores were as follows: 26.6% (n = 216) scored 0 points, 9.7% (n = 79) scored 1 point, and 8.4% (n = 68) scored 2 points. Overall, 63.7% (n = 517) scored 2 points or more, indicative of poor adherence. The highest value was 61 points, recorded by 1 respondent.

### Non-adherence score correlations

Table 2 displays the results of the Kruskal-Wallis tests and pairwise comparisons of non-adherence scores for each category within the independent variables. Statistically significant differences in non-adherence scores were found across three age categories showing lower non-adherence score (i.e. better adherence) associated with higher age levels. Respondents born and raised in Norway showed better adherence than respondents born abroad in the Kruskal Wallis test; however, these differences were not statistically significant after applying the Bonferroni correction. Respondents with master’s degree showed significantly better adherence compared to respondents with high school degree. Across categories of “years of regular medication use,” significantly better adherence was observed among individuals who had been using medications for 6-9 years, or 10 years or more, compared to those who did not use medications regularly. Furthermore, using 5-9 medications daily was associated with significantly better adherence compared to not using medications daily, and the use of a pill organiser was also linked to significantly better adherence compared to not using one. For the anchor-question better adherence was associated with strong beliefs in one’s own adherence. Additionally, significant differences in non-adherence scores emerged across the SDM-question categories, with improved adherence being associated with a heightened perception of inclusion in decision-making. Notably, gender was excluded from the study’s comparisons due to a highly skewed gender distribution. Furthermore, significant differences in non-adherence scores were found when comparing the MHD, pain and CVD subgroups, highlighting better adherence associated with CVD in comparison to pain and MHD.

**Table 2.** Pairwise comparisons of categories within variables with significant impact on adherence, based on Kruskal-Wallis tests. Total non-adherence scores as dependent variable. For the pairwise comparisons, Bonferroni corrections have been used, and both original and adjusted p-values are provided. For the adjusted *p*-values, significant values are marked with *.

Independent variables	Categories	n	Median non-adherence score	Pairwise comparisons of median non-adherence scores for the categories of independent variables Results only for when unadjusted p≤ .05	***P*-value**	**Adj. p-value**
Age	Young (18-44 years)	303	6	Young vs Middle aged Young vs Young-old Middle aged vs Young-old	<0.001	0.000*
	Middle aged (45-65 years)	404	0		<0.001	0.000*
	Young-old (66-79 years)	99	3		<0.001	0.000*
	Old-old (80-89 years)	6	4			
Born and raised	In Norway	748	3	Born and raised in Norway vs Born abroad, but lived more than eight years between ages 0 and 18 in Norway		
	Born abroad, but lived more than eight years between ages 0 and 18 in Norway	30	7.5		0.021	0.128
	Abroad	33	5			
	NA^a^	1	20			
Education	No education	7	9	No education vs Master Primary school vs Master High school vs Master Bachelor vs Master	0.015	0.226
	Primary school only	81	3		0.035	0.530
	High school or equivalent	425	4		0.002	0.037*
	Bachelor’s degree or equivalent	193	3		0.018	0.276
	Master’s degree or equivalent	96	2			
	NA^a^	10	2			
Number of selected medical condition groups	1	164	3	The o-value for the test statistic was not significant (α=0.05) and therefore pairwise comparisons were not performed		
	2	184	3			
	3	163	3			
	4	102	5			
	5-9	185	4			
	10 or more	14	5.5			
Years of regular medication use	0-1	53	4	0-1 years vs Not using regularly 2-5 years vs Not using regularly 6-9 years vs Not using regularly 10 years or more vs Not using regularly Not using regularly vs NA	0.027	0.406
	2-5	146	4		0.017	0.261
	6-9	109	3		<0.001	0.009*
	10 or more	444	3		<0.001	0.004*
	Not using regularly	49	5			
	NA^a^	11	1		0.025	0.370
Number of daily medications	0	52	4.5	0 vs 2 medications 0 vs 3 medications 0 vs 4 medications 0 vs 5-9 medications 0 vs 10 or more medications 1 vs 4 medications 1 vs 5-9 medications 1 vs 10 or more medications	0.008	0.221
	1	146	5		0.044	1.000
	2	150	3		0.005	0.127
	3	138	4		0.002	0.043*
	4	97	3		0.004	0.109
	5-9	166	2		0.036	1.000
	10 or more	55	2		0.011	0.295
	NA^a^	8	6		0.029	0.822
Using a pill organiser	Yes	319	2	Yes vs No	0.01	0.03*
	No	485	4			
	NA^a^	8	4			
Anchor-question	Very limited extent	5	11	Very limited vs Very large Limited vs Large Limited vs Very large Large vs Very large Limited vs NA	0.046	0.459
	Limited extent	24	21		<0.001	0.009*
	Large extent	206	10		<0.001	0.000*
	Very large extent	574	2		<0.001	0.000*
	NA^a^	3	1		0.007	0.071
SDM-question	Very limited extent	20	16.5	Very limited vs Large Very limited vs Very large Limited vs Large Limited vs Very large Large vs Very large Large vs NA Very large vs NA	0.004	0.040*
	Limited extent	84	11		<0.001	0.000*
	Large extent	269	5		<0.001	0.000*
	Very large extent	418	2		<0.001	0.000*
	NA^a^	21	7		<0.001 0.027 <0.001	0.000* 0.265 0.000*
Non-adherence score for medical condition groups	Pain group	191	6	Pain group vs CVD-group MHD-group vs CVD-group	<0.001	0.000
	Mental Health Disorders (MHD)-group	44	6		<0.001	0.000
	Cardiovascular Diseases (CVD)-group	117	0			

a NA; Not applicable.

### Non-adherence score and medical conditions for medication use

Results for reported medical condition groups for medication use and their total non-adherence scores are listed in Table 3. Respondents using medication for substance abuse problems or obstetrical disorders had the highest total non-adherence scores, indicating the poorest adherence. However, the number of respondents in each of these subgroups was insufficient for statistical analysis. Conversely, eye disorders and diseases, CVD and cancer scored the lowest total non-adherence scores, indicating the best adherence to medication.

**Table 3.** Total non-adherence scores for the medical condition groups.

Medical condition groups	Median of total non-adherence score	Inter-quartile Range Q1-Q3	*n* (%)
Total sample	3^a^	0-10	812 (100)
Substance abuse problems	28	-^b^	2 (0.3)
Obstetrical disorders	13.5	3.8-16.8	8 (1.0)
Fever, nausea, vomiting, dizziness, travel and motion sickness, hiccups, restless legs, leg cramps, etc.	7	2-12.8	76 (9.4)
Upper respiratory tract & otorhinolaryngologic disorders	6	2-14	86 (10.6)
Palliative care	5	0-14	26 (3.2)
Pain	5	1-12	365 (45.0)
Musculoskeletal disorders	5	1-11	239 (29.4)
Mental health disorders	5	1-11	154 (19.0)
Gynaecological disorders & contraception	5	2-10	95 (11.7)
Lower respiratory tract diseases	4	0-12	143 (17.6)
Infectious diseases	4	0-10	75 (9.2)
Allergies	4	1-11	295 (36.3)
Sleep related disorders	4	1-10	214 (26.4)
Gastrointestinal disorders	4	0-9	197 (24.3)
Dermatological disorders	4	1-10.3	114 (14.0)
Nervous system diseases	4	1-9	35 (4.3)
Kidney and urinary tract disorders	3.5	1-9	34 (4.2)
Other	3	0.5-9.5	61 (7.5)
Prostate problems	2.5	0-17.8	4 (0.5)
Immune system malfunctions & transplants	2	0-9	67 (8.2)
Blood related disorders	2	0-7	33 (4.1)
Endocrine diseases	2	0-6.3	126 (15.5)
Cardiovascular diseases	1	0-5	257 (31.7)
Cancer	1	0-5	18 (2.2)
Eye disorders and diseases	1	0-4	23 (2.8)
Do not know/do not want to tell/not applicable	15.5	-^b^	2 (0.3)

a Mean 6.7 (SD 8.8).

b Only 2 respondents.

### Quantifying causes of non-adherence

**Figure 2.**
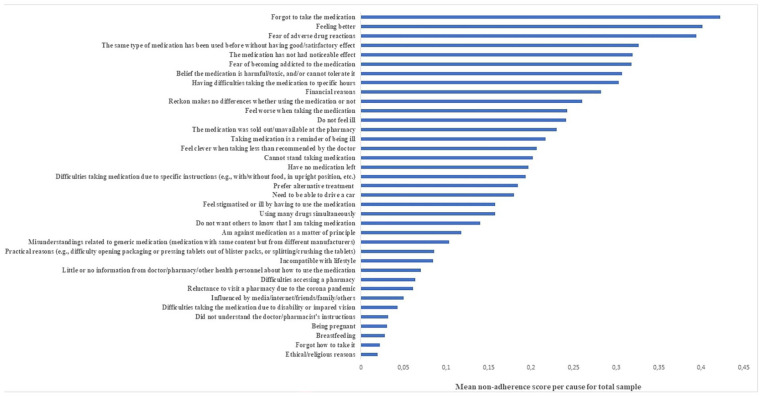
Mean non-adherence score for each cause of non-adherence for total sample, n = 812. Based on a non-adherence scale where: “very often” = 3, “often” = 2, sometimes” = 1,“rarely/never” = 0.

Figure 2 displays the mean non-adherence scores for the 37 causes of non-adherence in OMAS-37, where the most substantiated causes of poor adherence are *Forgetting to take the medication* in first place, *Feeling better* in second, and *Fearing adverse drug reactions* in third. Conversely, *Forgetting how to use the medication* and *Ethical or religious reasons* registered the lowest scores, indicating less influential causes of poor adherence.

When comparing the top five causes among the subgroups, *Forgetting to take the medication* held the highest rank in both the MHD-group and the CVD-group, while it was fourth in the pain group. *Fearing adverse drug reactions* ranked highest in the pain group. *Feeling better* was second in the pain group and third for the MHD-group but is not among the top five for the CVD-group. *Fearing adverse drug reactions* was second in the CVD-group and fourth in the MHD-group. *Using the same type of medication before without satisfactory effect* was second in the MHD-group, fourth in the CVD-group and fifth in the pain group. *Having difficulties taking the medication at specific hours* was fifth in the MHD-group but was not among the top five in the two other groups. The CVD-group ranked *Feeling medications are harmful/toxic, and/or not tolerating them* as third, a cause that was not present in the top five for the other two groups.

## Discussion

This is the first study to employ OMAS-37 on a large scale which includes patients facing various medical challenges. The study revealed that nearly 64% of the respondents demonstrated suboptimal medication adherence. Factors such as increasing age, higher education, involvement in medication decision, and utilisation of pill organisers were found to have a statistically significant positive association with adherence. However, the degree of non-adherence differed across various medical conditions. The main causes for non-adherence were forgetfulness, perceived improvement in health, and fear of adverse drug reactions. Nonetheless, there were certain variations in the main causes of non-adherence among the total sample and three selected patient subgroups—those using medication for cardiovascular diseases, pain management, or mental health disorders.

### Strengths and limitations

OMAS-37 is a non-disease specific tool and provides a versatile means to assess adherence in varied populations. It quantifies both adherence levels and main causes of non-adherence.

A limitation for this study is the lack of power calculations, as this study represents the first use of OMAS-37. However, efforts have been made to include a large sample size, and alternative methods for determining adequate sample size confirm that a sample size of 812 is considered large enough to draw meaningful conclusions.^17^ The e-survey collected anonymous responses without utilising IP-addresses or cookies, which may introduce bias due to the potential for multiple submissions by respondents. Recruitment through Facebook has limitations, particularly in calculating view and response rates due to the Facebook algorithm, as noted in the OMAS-37 validation paper.^15^ Additionally, the membership compositions of the recruited Facebook groups / pages could have influenced the study’s outcomes. Both the recruitment method and the skewed gender distribution in this study introduces a bias that limits the generalizability of the results to the broader population. Several factors may explain why women are often overrepresented in online health surveys.^18^ On average, women tend to show greater interest in health and wellness topics, whereas men may perceive health-related questionnaires as less relevant. Women also typically engage more actively on social media platforms.^19^ Furthermore, social media algorithms might contribute to this imbalance by promoting health-related content more frequently to women based on their online behaviour.

About 80% of respondents reported multiple medical conditions, implying that data from a single respondent could impact the results for several conditions and introduce potential bias. In comparing non-adherence scores among the three patient subgroups pain, CVD and MHD, the overlapping one third of respondents who had multiple of these medical conditions were excluded from the between-groups analysis, which may contribute to bias. Lastly, the respondents were utilising one or more medications, but the specific adherence phase—initiation, implementation, and persistence—for each medication was not specified. This makes any potential impact of the adherence phase difficult to assess.^20^

### Adherence levels and associations with education, age, and medication count

Among the respondents, 64% exhibited scores indicating poor adherence, in line with the known variability of adherence levels.^2,21^ Improved adherence was associated with individuals with a master’s degree, which aligns with previous studies indicating the positive influence of higher education on adherence.^2,6^ The findings indicated poorer adherence among younger age groups, which is consistent with recent systematic reviews that show better adherence in middle and older age groups, and poorer adherence observed in both very young and very old individuals.^6,22^ One possible explanation is that older adults may have better routines and more experience as medication users. These results could also be related to the observation that using more medications daily was associated with enhanced adherence. The conventional assumption is that polypharmacy contributes to non-adherence because of the increased potential for missing doses when managing multiple medications. However, our observation that using five to nine medications daily was significantly associated with better adherence compared to not using medications daily aligns with a study by Reach et al.^23^ They reported that having more than seven medications was associated with full adherence. Approximately 40% of the respondents reported using a pill organiser, and the findings indicated better adherence among those using such devices. It remains uncertain whether the positive association between adherence and number of medications is due to the use of pill organisers. Furthermore, it is uncertain whether the improved adherence among those using pill organisers is due to the pill organisers themselves, or if these organisers are primarily adopted by individuals who are already proactive in maintaining adherence and are more aware of the severity of their disease. Some literature aligns with this result and suggests limited evidence for the effectiveness of pill organisers in enhancing adherence.^24^

### Adherence levels and shared decision making

This study also demonstrated a positive association between adherence and feeling involved in medication decision-making (SDM-question), which is consistent with literature on SDM.^25,26^ The large proportion of respondents (85%) feeling highly involved in their medication treatment decisions aligns with the prioritised implementation of SDM at all healthcare levels in Norway.^27^ However, although the non-adherence score indicated that approximately 64% had poor adherence, the anchor-question revealed that 96% believed they were following the doctor’s recommendations to a “large extent” or “very large extent.” This could be attributed to the tendency for people who respond to medication questionnaires to be more engaged with their medication regime, resulting in overestimations of self-reported adherence.^28^ *Forgetting to take medication*, *feeling better* and *fearing adverse drug reactions* are well known causes of non-adherence,^2,6,29^ which is consistent with the findings in this study.

### Adherence levels in selected diagnosis

This study found significantly better adherence within the CVD-group compared to the pain- and MHD-groups. CVD medications typically involve long-term use and may be prescribed to patients who may not experience noticeable physical symptoms. The findings of improved adherence within the CVD-group align with previous research on patients using cardiovascular medications, which frequently emphasises strong adherence.^9,10^ Pain medications are frequently used sporadically/for short-term relief by patients experiencing pain symptoms. In this study, the pain group demonstrated poor adherence consistent with a previous study.^30^ Our findings indicated better adherence among individuals using medication regularly, which supports the notion of reduced adherence in the pain group. The MHD-group was selected based on literature indicating that patients suffering from MHD are likely to be non-adherent.^6,31^ In this study the MHD-group displayed the poorest adherence among the 3 subgroups. Notably, the MHD-group was the sole group to report *Having difficulties taking the medication at specific hours* as one of the top five causes of non-adherence. Regarding the anchor-question, 80% of the CVD-group and 70% of both the MHD- and pain groups reported following their doctor’s recommendations to a “very large extent”. These findings align well with the distinct patterns observed in the total non-adherence scores for the CVD-group (3.7 points) versus the MHD-group (8.1 points) and the pain group (7.7 points).

In existing literature variations in methodology, demographic biases, and selective or incomplete result reporting may account for dissimilar conclusions regarding associations between background variables and adherence.^6,29^ Further studies will be necessary to determine whether the findings apply to the general Norwegian population, particularly considering the skewed gender distribution.

### Implications for policy, practice and research

Extensive poor adherence highlights the ongoing need for adherence-enhancing interventions. Such measures are crucial to enhance the likelihood of attaining the desired health outcomes, a significant aspect of the quality of care, as highlighted by the World Health Organization.^32^ To improve adherence in practice, healthcare providers should involve patients in treatment decisions and address medication adherence concerns also with younger patients and those with few prescribed medications. As feeling involved in decisions about medical treatment enhances adherence,^33^ increasing the number of patients who feel involved in medication decisions is crucial. Patients feel involved when they are listened to and understood. This study demonstrates that the causes of non-adherence vary across different patient groups, emphasizing the importance of understanding each patient’s main concerns. Addressing these concerns and considering them in the decision-making process is key to improving adherence.

Variations in the specific main causes of non-adherence across patient subgroups suggest the necessity of tailored interventions based on drug/medical condition. The OMAS-37 applies to all medication adherence phases and offers a novel pathway for supporting the development of adherence-enhancing guidance and interventions. Future research should explore the effectiveness of adherence-enhancing interventions tailored to the main causes of non-adherence within specific patient groups, as identified by OMAS-37.

## Conclusion

This study is the first to use OMAS-37 and demonstrates high levels of non-adherence both in the general population and among specific patient subgroups. Factors showing a statistically significant positive association with adherence included increasing age, higher education, involvement in medication decisions, and the use of pill organisers. Furthermore, the study elucidates that causes of non-adherence differ among distinct patient groups. These findings underscore the significance of implementing adherence-promoting interventions tailored to specific patient groups facing diverse adherence barriers. The efficacy of the OMAS-37-tool is highlighted, proving to be a valuable instrument for both assessing adherence and pinpointing causes of non-adherence for targeted interventions in future studies.

## Supplemental Material

sj-docx-1-inq-10.1177_00469580251321596 – Supplemental material for High Medication Non-Adherence Rates and its Drivers in the General Population: A Cross-sectional Study Using the OMAS-37 Adherence Survey Tool Supplemental material, sj-docx-1-inq-10.1177_00469580251321596 for High Medication Non-Adherence Rates and its Drivers in the General Population: A Cross-sectional Study Using the OMAS-37 Adherence Survey Tool by Rønnaug Eline Larsen, Ala Karimi, Tonje Krogstad, Cecilie Johannessen Landmark and Lene Berge Holm in INQUIRY: The Journal of Health Care Organization, Provision, and Financing
