# Supplementary material for: High Medication Non-Adherence Rates and its Drivers in the General Population: A Cross-sectional Study Using the OMAS-37 Adherence Survey Tool
Source: Inquiry. 2025 Feb 24;62:00469580251321596. doi: 10.1177/00469580251321596 (PMC11851734; doi:10.1177/00469580251321596)
Supplement: sj-docx-1-inq-10.1177_00469580251321596 – Supplemental material for High Medication Non-Adherence Rates and its Drivers in the General Population: A Cross-sectional Study Using the OMAS-37 Adherence Survey Tool [file sj-docx-1-inq-10.1177_00469580251321596.docx]

**Supplementary file - The e-survey in English**

This supplementary file is an AI translation of the Norwegian survey. The translated items are not validated and should not be used in its current English form. Please contact the author team for a validated translated version.

**Questionnaire on Medication Use**

Thank you for taking the time to complete this questionnaire.

This survey is conducted by OsloMet – Oslo Metropolitan University, in collaboration with other partners. The questionnaire is completely anonymous and takes approximately 10 minutes to complete. Please answer the questions as honestly as possible – your responses are anonymous. If you are unsure about how to answer a question, choose the option you feel fits best. By completing and submitting the questionnaire, you consent to participate in the survey.

The questionnaire consists of three main sections: a section with background questions, a section with specific questions, and finally a brief concluding section.

Your response is very valuable to us. Thank you so much for your participation.

**Part 1: Background questions**

1. Are you 18 years or older?

- Yes
- No

**1a) What is your age**

*(This item is only displayed if the option "Yes" is selected for the question "1) Are you 18 years old or older?")*

- Dropdown-meny

1. **What is your gender?**

*(This item is only displayed if the option "Yes" is selected for the question "1) Are you 18 years old or older?")*

- Man
- Woman
- Don't know / Not applicable / Prefer not to answer

1. **What is your highest level of completed education?**

*(This item is only displayed if the option "Yes" is selected for the question "1) Are you 18 years old or older?")*

- No education
- Primary school
- Upper secondary school / vocational school / high school
- University and college: Bachelor's degree or equivalent (3 years)
- University and college: Master's degree or equivalent (5 years), or higher
- Don't know / Not applicable / Prefer not to answer

Page shift

**4a) Do you live permanently in Norway? If you spend part of the year abroad and part of the year in Norway, please check "Yes."**

*(This item is only displayed if the option "Yes" is selected for the question "1) Are you 18 years old or older?")*

- Yes
- No
- Don't know / Not applicable / Prefer not to answer

**4b) Where were you born and raised?**

*(This item is only displayed if the option "Yes" is selected for the question "1) Are you 18 years old or older?")*

- Born and raised in Norway
- Born in another country, but spent the entire/majority of childhood in Norway (you have spent 8 years or more of the first 18 years of life in Norway)
- Born and raised in another country / countries
- Don't know / Not applicable / Prefer not to answer

**4c) Which country/countries? (Note: Do not provide personally identifiable information)**

*(This item is only displayed if the option "Born in another country, but spent the entire/majority of childhood in Norway (you have spent 8 years or more of the first 18 years of life in Norway)" or "Born and raised in another country / countries" is selected in the question "4b) Where were you born and raised?")*

- Textbox

**4d) How well do you think you understand Norwegian?**

*(This item is only displayed if the option "Born in another country, but spent the entire/majority of childhood in Norway (you have spent 8 years or more of the first 18 years of life in Norway)" or "Born and raised in another country / countries" is selected in the question "4b) Where were you born and raised?")*

- Very well
- Well
- Somewhat poorly
- Poorly
- Don't know / Not applicable / Prefer not to answer

Page shift

**Section 2: Treatment and Health Questions**

In the rest of the survey, we refer to medications that are prescribed or recommended by a doctor. This includes both prescription and over-the-counter medications.

**5) In the past 12 months, have you RECEIVED a prescription or been recommended to use medication by a doctor? (This applies to both prescription and over-the-counter medications)**

*(This item is only displayed if the option "Yes" is selected for the question "1) Are you 18 years old or older?")*

- Yes
- No
- Don't know / Not applicable / Prefer not to answer

**6) In the past 12 months, have you USED one or more medications prescribed or recommended by a doctor? (This applies to both prescription and over-the-counter medications)**

*(This item is only displayed if the option "Yes" is selected for the question "1) Are you 18 years old or older?")*

- Yes
- No
- Don't know / Not applicable / Prefer not to answer

**7) What did you answer to questions 5 and 6?**

*(This item is only displayed if the option "Yes" is selected for the question "1) Are you 18 years old or older?")*

- Answered YES to both
- Answered NO to both
- Answered NO to question 5 and YES to question 6
- Answered YES to question 5 and NO to question 6
- Answered "Don't know / Not applicable / Prefer not to answer" to question 5 and/or 6

**7aYou are receiving this question because you answered YES to question 5 and NO to question 6. You have stated that you have received a prescription or recommendation for medication from a doctor, but have not used the medication(s) in the past 12 months. What is the reason you have chosen not to use the medication(s) you were recommended? (Note: Do not provide personally identifiable information)**

*(This item is only displayed if the option "Answered YES to question 5 and NO to question 6" is selected in the question "7) What did you answer to questions 5 and 6?")*

- Textbox

**8) For which diagnosis(es) have you used medication for in the past 12 months?**

*(This item is only displayed if the option "Answered YES to both," "Answered 'Don't know / Not applicable / Prefer not to answer to question 5 and/or 6," or "Answered NO to question 5 and YES to question 6" is selected in the question "7) What did you answer to questions 5 and 6?")*

Please check all relevant diagnoses.

- Have not used medication in the past 12 months
- Cardiovascular diseases (e.g. high blood pressure, blood thinners, heart failure, stroke, heart attack, angina, arrhythmia, high cholesterol, etc.)
- Pain (e.g. caused by injury/accident, headaches, muscle/skeletal/nerve pain, etc.)
- Blood disorders (e.g. blood clots, anemia, increased tendency to bleed, etc.)
- Musculoskeletal diseases (e.g. osteoporosis, arthritis, etc.)
- Mental health disorders (e.g. anxiety, depression, psychosis, schizophrenia, obsessive-compulsive disorder (OCD), bipolar disorder, phobias, etc.)
- Sleep problems
- Substance use problems
- Endocrine disorders (e.g. diabetes, thyroid issues, etc.)
- Gastrointestinal disorders (e.g. ulcers, heartburn, acid reflux, inflammatory owel disease, constipation, diarrhea, etc.)
- Upper respiratory, ear, mouth, nose, and throat diseases (e.g. sinusitis, chronic nasal congestion, nosebleeds, dry mouth, dental issues, snoring, etc.)
- Lower respiratory diseases (e.g. asthma, COPD, cough, etc.)
- Immune system disorders and transplants (including autoimmune diseases, etc.)
- Kidney and urinary tract diseases (e.g. kidney failure, kidney stones, urinary incontinence, urinary retention, erectile dysfunction, etc.)
- Cancer (e.g. leukemia/blood cancer, prostate cancer, breast cancer, etc.)
- Prostate issues
- Infectious diseases (e.g. urinary tract infections, flu, skin infections, cold sores, HIV and AIDS, tuberculosis, sexually transmitted infections, etc.)
- Skin, hair, and nail disorders (e.g. eczema, psoriasis, acne, hair disorders, nail fungus, etc.)
- Neurological diseases (e.g. epilepsy, Parkinson’s disease, movement disorders, multiple sclerosis, etc.)
- Eye diseases (e.g. glaucoma, cataracts, etc.)
- Allergies (e.g. to animals, pollen, cosmetics, food, etc.)
- Gynecological diseases and contraception (e.g. menstrual and bleeding disorders, contraception, infertility, etc.)
- Pregnancy, childbirth, and breastfeeding (e.g. nausea, high blood pressure, edema (water retention), epilepsy during pregnancy, low milk production, sore nipples, weak milk ejection reflex, etc.)
- Fever, nausea, vomiting, dizziness, motion sickness, hiccups, restless legs, leg cramps, etc.
- Palliative care (treatment not intended to cure a disease)
- Other
- Don't know / Not applicable / Prefer not to answer

**8a)** **If you selected "Other," you can specify it here (Note: Do not provide personally identifiable information):**

*(This item is only displayed if the option "Other" is selected in the question "8) For which diagnosis(es) have you used medication for in the past 12 months?")*

- Textbox

**9) Do you take medication regularly?**

*(This item is only displayed if the option "Answered YES to both," "Answered 'Don't know / Not applicable / Prefer not to answer to question 5 and/or 6," or "Answered NO to question 5 and YES to question 6" is selected in the question "7) What did you answer to questions 5 and 6?")*

- Yes
- Only during certain periods (e.g. pollen allergy)
- No
- Don't know / Not applicable / Prefer not to answer

**9a) How long have you been taking medication regularly? (This also applies to intermittent use, e.g. for pollen allergy)**

*(This item is only displayed if the option "Don't know / Not applicable / Prefer not to answer," "Only during certain periods (e.g. pollen allergy)," or "Yes" is selected in the question "9) Do you take medication regularly?")*

- 0 - 1 year
- 2 - 5 years
- 6 - 9 years
- 10 years or more
- Don't know / Not applicable / Prefer not to answer

**10) How many medications do you usually take daily?**

*(This item is only displayed if the option "Answered YES to both," "Answered 'Don't know / Not applicable / Prefer not to answer to question 5 and/or 6," or "Answered NO to question 5 and YES to question 6" is selected in the question "7) What did you answer to questions 5 and 6?")*

- 0
- 1
- 2
- 3
- 4
- 5-9
- 10 or more
- Don't know / Not applicable / Prefer not to answer

**11) Are you primarily responsible for taking your medications?**

*(This item is only displayed if the option "Answered YES to both," "Answered 'Don't know / Not applicable / Prefer not to answer to question 5 and/or 6," or "Answered NO to question 5 and YES to question 6" is selected in the question "7) What did you answer to questions 5 and 6?")*

- Yes
- No
- Don't know / Not applicable / Prefer not to answer

Page shift

Below are a number of common reasons why some people do not take their medications as recommended by their doctor. For each of these reasons, we would like you to check how often you do not follow your doctor's recommendations regarding the use of your medications.

Not following your doctor's recommendations means, for example, forgetting, choosing not to take the medication, taking less or more than prescribed, or taking it in a different way.

**12a) How often do you fail to follow your doctor's recommendations regarding the use of your medications?**

*(This item is only displayed if the option "Answered YES to both," "Answered 'Don't know / Not applicable / Prefer not to answer to question 5 and/or 6," or "Answered NO to question 5 and YES to question 6" is selected in the question "7) What did you answer to questions 5 and 6?")*

*All sub-questions here should be answered with one of the following options:*

- *Very often*
- *Often*
- *Occasionally*
- *Rarely / Never*

**Patient-specific reasons:**

- Because you think it’s not that important whether you take your medications or not
- Because you forgot
- Because you ran out of medication
- Because you forgot how to use it
- Because you didn’t understand what the doctor or pharmacist meant
- Because you feel good when you take less than the doctor recommended
- Because you are afraid of side effects
- Because you feel the medications are harmful, toxic, or you can’t tolerate them
- Because you are afraid of becoming dependent on the medications
- Because you can’t bring yourself to take the medications
- Due to practical reasons (e.g. it’s difficult to open the medication box, press out the pills, or divide/crush the tablet)
- Due to disability (e.g. it’s difficult to swallow the pill, or impaired vision that makes it hard to find the right medication)
- Due to the need to drive
- Because you are pregnant
- Because you are breastfeeding
- Because you feel stigmatized or pathologized by having to use medications
- Because you don’t want others to know that you are using medications
- Because you are fundamentally opposed to treatment with medications
- Because you prefer alternative treatments
- Because taking medications doesn’t fit your lifestyle
- Because you don’t want to be sick, and taking medications reminds you of this

Page shift

**12b) How often do you fail to follow your doctor's recommendations regarding the use of your medications?**

*(This item is only displayed if the option "Answered YES to both," "Answered 'Don't know / Not applicable / Prefer not to answer to question 5 and/or 6," or "Answered NO to question 5 and YES to question 6" is selected in the question "7) What did you answer to questions 5 and 6?")*

*All sub-questions here should be answered with one of the following options:*

- *Very often*
- *Often*
- *Occasionally*
- *Rarely / Never*

**Therapy-related reasons:**

- Because you have used the same type of medication before without it being effective
- Because you are taking many medications at the same time
- Because you have difficulty taking your medications at specific times
- Because you have difficulty taking your medications under specific conditions (e.g. with or without food, in an upright position, etc.)

Page shift

**12c) How often do you fail to follow your doctor's recommendations regarding the use of your medications?**

*(This item is only displayed if the option "Answered YES to both," "Answered 'Don't know / Not applicable / Prefer not to answer to question 5 and/or 6," or "Answered NO to question 5 and YES to question 6" is selected in the question "7) What did you answer to questions 5 and 6?")*

*All sub-questions here should be answered with one of the following options:*

- *Very often*
- *Often*
- *Occasionally*
- *Rarely / Never*

**Condition- or disease-related reasons:**

- Because you feel better
- Because you feel worse when you take them
- Because you don’t feel sick
- Because you don’t notice any effect from the medications

Page shift

**12d) How often do you fail to follow your doctor's recommendations regarding the use of your medications?**

*(This item is only displayed if the option "Answered YES to both," "Answered 'Don't know / Not applicable / Prefer not to answer to question 5 and/or 6," or "Answered NO to question 5 and YES to question 6" is selected in the question "7) What did you answer to questions 5 and 6?")*

*All sub-questions here should be answered with one of the following options:*

- *Very often*
- *Often*
- *Occasionally*
- *Rarely / Never*

**Health system- and healthcare-related reasons:**

- Because the medications were out of stock or unavailable at the pharmacy
- Due to little or no information from the doctor, pharmacist, or other healthcare personnel about how to use your medications
- Due to misunderstandings related to generic medications (medications with the same active ingredient, but from different manufacturers)

Page shift

**12e) How often do you fail to follow your doctor's recommendations regarding the use of your medications?**

*(This item is only displayed if the option "Answered YES to both," "Answered 'Don't know / Not applicable / Prefer not to answer to question 5 and/or 6," or "Answered NO to question 5 and YES to question 6" is selected in the question "7) What did you answer to questions 5 and 6?")*

*All sub-questions here should be answered with one of the following options:*

- *Very often*
- *Often*
- *Occasionally*
- *Rarely / Never*

**Social and economic reasons:**

- Due to financial reasons
- Due to ethical or religious reasons
- Because it is difficult for you to get to a pharmacy
- Because you don’t want to go to the pharmacy due to the COVID-19 pandemic
- Because you have been influenced by media, the internet, friends, family, and/or others

Page shift

12f) If you have any comments or other reasons, you can write them here (Please do not provide personally identifiable information):

*(This item is only displayed if the option "Answered YES to both," "Answered 'Don't know / Not applicable / Prefer not to answer to question 5 and/or 6," or "Answered NO to question 5 and YES to question 6" is selected in the question "7) What did you answer to questions 5 and 6?")*

- Textbox

Page shift

**Part 3: Closing Questions**

**13) Overall, to what extent do you believe you follow the doctor's recommendations regarding the use of your medications?**

*(This item is only displayed if the option "Answered YES to both," "Answered 'Don't know / Not applicable / Prefer not to answer to question 5 and/or 6," or "Answered NO to question 5 and YES to question 6" is selected in the question "7) What did you answer to questions 5 and 6?")*

- To a very small extent
- To a small extent
- To a large extent
- To a very large extent
- Don’t know / Not applicable / Prefer not to answer

**14) To what extent do you feel included in decisions regarding your medication treatment?**

*(This item is only displayed if the option "Answered YES to both," "Answered 'Don't know / Not applicable / Prefer not to answer to question 5 and/or 6," or "Answered NO to question 5 and YES to question 6" is selected in the question "7) What did you answer to questions 5 and 6?")*

- To a very small extent
- To a small extent
- To a large extent
- To a very large extent
- Don’t know / Not applicable / Prefer not to answer

**15) Finally: Do you use a pill organizer? (See example of a pill organizer in the image below)**

*(This item is only displayed if the option "Answered YES to both," "Answered 'Don't know / Not applicable / Prefer not to answer to question 5 and/or 6," or "Answered NO to question 5 and YES to question 6" is selected in the question "7) What did you answer to questions 5 and 6?")*

- Yes
- No
- Don’t know / Not applicable / Prefer not to answer

**Is there anything you would like to add? Please feel free to use the comment section below. We are happy to receive general feedback, suggestions, and comments. (Please do not provide personally identifiable information).**

*(This item is only displayed if the option "Answered YES to both," "Answered 'Don't know / Not applicable / Prefer not to answer to question 5 and/or 6," or "Answered NO to question 5 and YES to question 6" is selected in the question "7) What did you answer to questions 5 and 6?")*

- Textbox

**Thank you for participating in our survey by answering this questionnaire. We greatly appreciate your contribution. If, after responding to questions about medication use and/or completing the patient education section, you feel uncertain or have any questions about your medications, we recommend that you contact your doctor or ask at the pharmacy.**

**You have indicated that you are under 18 years old, and therefore, you are not part of the target group for this survey. Thank you for your willingness to participate. If this was an error and you are over 18, you can go back by clicking "Previous page."**

*(This item is only displayed if "No" is selected in the question "1) Are you 18 years or older?")*

**Thank you for your participation in the survey. Since you indicated that you have neither received a prescription nor been recommended to use medication by a doctor, and that you have not used medication in the past 12 months, there are no further questions. We truly appreciate your contribution.**

*("This item is only shown if the option 'Answered NO to both' was selected in question '7) What did you answer to questions 5 and 6?'")*

**Press send to finish.**
